# How to calculate the life cycle of high-risk medical devices for patient safety

**DOI:** 10.3389/fpubh.2022.989320

**Published:** 2022-09-14

**Authors:** Gihong Seo, Sewon Park, Munjae Lee

**Affiliations:** ^1^Medical device & Healthcare Solution (MEDIPLY), Seoul, South Korea; ^2^Department of Medical Humanities and Social Medicine, Ajou University School of Medicine, Suwon, South Korea; ^3^Medical Research Collaborating Center, Ajou Research Institute for Innovative Medicine, Ajou University Medical Center, Suwon, South Korea

**Keywords:** medical device, product lifecycle management (PLM), service life, medical device regulation, patient safety

## Abstract

In this study, we analyzed Korean and foreign systems, focusing on high-risk medical devices that urgently need to be managed, and we present an life cycle calculation method for determining replacement time. A literature review was conducted to confirm the regulations of the medical device management system and life cycle by country, and a case analysis was performed to verify the replacement evaluation criteria of actual medical institutions. In addition, durability data from the Public Procurement Service, American Hospital Association, and Samsung Medical Center were used to calculate the life cycle of high-risk medical devices. The analysis showed that in the case of Korean and foreign medical device regulatory agencies, there were no specific life cycle regulations for high-risk medical devices. In addition, the important items in the medical device replacement evaluation were found to be the year of introduction, repair cost, component discontinuation, and several failures. On calculating the life cycle of high-risk medical devices revealed that the replacement time is 13 years for anesthesia machines, 14 years for defibrillators, 16 years for heart-lung machines, and 13 years for ventilators. To introduce a uniform medical device replacement standard and life cycle calculation method, the government will need to reorganize the medical device replacement laws and systems. In addition, in the case of medical institutions, it is necessary to secure patient safety by using expert groups to prepare specific life cycle standards that consider the characteristics of medical devices.

## Introduction

Medical devices are used to diagnose or treat patients, and they have direct or indirect effects on human life and health. For this reason, safety is paramount during their use, and it is necessary to prevent downtime and maintenance costs due to unexpected shutdowns. Accordingly, considerable effort is required to plan and manage the purchase, use, maintenance, and replacement of medical devices (1). In particular, to provide optimal medical services to patients, the periodic replacement of medical devices should be fulfilled at the right time in accordance with established standards and procedures. These medical device replacement methods include use -period assesment according to the purchase year, device replacement based on use-period and budget, and establishing an evaluation system for replacement and extracting the data by rank according to evaluation items (2, 3).

For all products, a replacement process takes placewhere they are discarded after a certain period of time and then a new product takes their place. This is called the product life cycle, and it is unique to each product (4, 5). Medical devices also have life cycles; and unlike industrial products, are key in the functioning of human life. Therefore, they should be managed in accordance with strict laws and regulations in the processes of design, production, distribution, and sales. In particular, when they are used directly on the human body in a medical institution after sale, special attention is required for the performance and safety of the product. To manage this, each country has regulatory agencies to manage medical devices in accordance with established laws and regulations (6, 7).

Medical devices can be sold after approval by regulatory agencies for their safety and performance suitability. In particular, in the licensing process for medical devices, problems such as performance degradation and failures, which may occur depending on the period used, are also considered. As aged medical devices are more likely to malfunction or show errors during treatment, they must be managed to determine whether the device is being used safely, and the target performance must be maintained through regular performance and safety inspections. However, as the number of patients suffering from old medical device failures increases, managing the life cycles, the period during which medical devices can be used, is required. The safety of patients can be secured by controlling the risks associated with the period of use of the medical device, and reducing the side effects (8, 9).

In the case of medical devices utilized in Korean medical institutions, document inspections are conducted once a year for special medical equipment and those that use imaging are closely inspected every 3 years to manage the image quality. Diagnostic radiation generators have been regularly managed every 3 years by legislation for regular inspections of radiation safety. Emergency equipment, including automated external defibrillators (AEDs), must be inspected at least once a month in accordance with the Emergency Medical Service Act. However, the current medical law stipulates the requirements on the image quality, performance, safety, and inspection intervals for only 16 types of special medical equipment, diagnostic radiation generators, etc., but no recommendations on replacement timing or any specific regulations on life cycle for other medical devices. Medical devices are used to provide safer and more accurate diagnoses and treatments for patients; however, such a gap in legal regulations can lead to performance deterioration of medical devices, causing an increased risk to patients (10).

To manage medical devices, medical institutions classify them into high-risk, mid-risk, and low-risk devices. In particular, life support systems, such as respirators, anesthetic equipment, cardiopulmonary bypass machines, and surgical equipment, are directly related to the patient's life and are thus classified as high-risk medical equipment. In the case of these high-risk medical devices, voluntary management by medical institutions is implemented, so even if the same product is used, the use period is different for each medical institution, and the risk to patients increases due to device aging. Meanwhile, the use of artificial intelligence (AI) medical devices is expanding to provide personalized medical care and realize precision in medicine. An AI medical device, which is a software-based medical device, is considered high-risk because the operation of the software can be directly impact the patient, and every part of the product life cycle requires management to ensure performance and safety. As such, it is necessary to manage the performance and safety of the device through clear regulations on the life cycle of medical devices; however, the calculation methods of life cycles are different for each country and medical institution, and related research is lacking. Therefore, in this study, we compare and analyze domestic and foreign systems and regulations regarding the replacement time, centered on high-risk medical devices that urgently need management by priority, and then suggest calculation methods for life cycles to determine replacement times. Baseline data to manage the life cycle of high-risk medical devices should be provided, resulting in the establishment of a medical system that can provide medical practices that ensure patient safety.

## Materials and methods

This study proposes life cycles for high-risk medical devices currently used in medical institutions. Our research was performed according to the following steps: First, a literature review was conducted to confirm the regulations governing the medical-device management system and life cycle by country. To this end, the United States, Japan, the United Kingdom, Canada, and Korea were selected based on the market size of the medical device industry in 2021. Through a review of academic papers, policy data, etc., an objective review was performed on the current status of medical device management systems for each country and each medical institution (11, 12). Second, 10 domestic and foreign medical institutions were selected, for which a case analysis was conducted to confirm the evaluation criteria for medical device replacement and derive major evaluation items. In addition, actual medical device replacement cases were identified at three higher medical institutions in South Korea, and an additional on-site investigation was carried out to check whether the life cycle was reflected in the replacement item, whether there was a regulation on the life cycle, etc. Third, to calculate the life cycle of high-risk medical devices, we analyzed the period in which medical devices were replaced at the Samsung Medical Center from 2009 to 2019, for which a comparative analysis was performed with the life cycle criteria of the American Hospital Association and the Korean Public Procurement Service.

### Life cycle in medical devices

The life cycle of medical devices — the period during which the medical device can be used while maintaining the state of the technical inspection certificate — refers to the life expectancy of the device that can be used for being used for treatment and inspection. The lifespan of a medical device is determined by various factors, in which representative factors include function, reliability, availability, and maintainability. The life cycle of a medical device is the usage limit that may meet the target values of these factors and guarantees safety. If the usage period is specified in the device specifications of the manufacturer, the usage limit will follow the standards (13).

Specifically, the life cycle of medical devices is divided into physical, commonplace, and economic life. Physical life refers to the state in which the malfunction of the device exceeds the tolerance limit due to the high frequency of failures caused by wear, corrosion, damage, etc. Commonplace life refers to the state in which it is advantageous to replace the device with a new one because the function has deteriorated due to technological advancement; there is a tendency for rapid technological advancement to shorten equipment lifespan. Economic life refers to the minimum point of average total cost as the life cycle, which is the sum of the average maintenance cost and average capital cost during the life cycle of a medical device (14). Of these, physical life is the longest, economic life is at the middle level, and commonplace life is the shortest.

### Management of high-risk medical devices

According to the Joint Commission (TJC), which approves medical institutions and programs in the United States, high-risk medical devices include life-support systems, and their failure could cause serious injuries or death to patients or related personnel. High-risk medical devices defined by TJC include heart-lung machines, ventilators, defibrillators, and robotic assistive devices (15, 16). All facilities with high-risk medical devices must implement the activities necessary for their maintenance, inspection, and testing of medical devices in accordance with the manufacturer's recommendations. According to the alternative equipment maintenance (AEM) program of medical devices, planned maintenance activities for high-risk medical devices must be 100% complete.

Meanwhile, the Korean healthcare accreditation system specifies the contents and purpose of medical institution accreditation under Article 58 of the Medical Law; the details are described in section 11.5, Medical Device Management of Chapter 3 in the Organizational Management System in the Accreditation Criteria Guidebook of the Korea Institute Healthcare Accreditation. The purpose of investigating evaluation accreditation is to perform continuous maintenance so that the medical device can operate accurately and in a timely manner and to create an environment to prevent malfunctions and enable safe medical services to be provided. A person with certain qualifications or licenses must be appointed as the person in charge of medical device safety management, who should manage a list of medical devices and specify the inspection method and interval for each list item. In addition, they should regularly update the list and separately manage the list of high-risk medical devices according to the degree of risk at the medical institution level. Based on this, preventive inspections, such as daily inspection and regular inspection, should be performed, and inspection confirmation labels listing the inspection department, implementation, and inspection date must be attached to the device. Furthermore, regular operational reports on the entire process of managing high-risk medical devices should be created for management.

### Life cycle calculation method

Methods to calculate the life cycle can be broadly divided into engineering and empirical methods. Investigating studies on the engineering calculation method of life cycle, Dondelinger (2004) stated that a replacement plan for medical devices should be established based on technical and economic feasibility (17). As the need for replacement will become more likely when a medical device breaks down, a large amount of accumulated data is required to consider the failure rate and repair costs, and a measure was suggested to calculate the appropriate life cycle based on the cumulative number of failures and repair costs.

On the other hand, examining the empirical life cycle calculation method, the Canadian Association of Medical Radiation Technologists (CAMRT) has published the life cycle guidelines for medical imaging devices to help determine when to upgrade or replace existing medical devices (18). In addition, the American Hospital Association (AHA) has proposed estimated figures of empirical life cycles, primarily for the purpose of accounting, and the American Society for Health Care Engineering (ASHE) has developed an empirical life cycle calculation method to support replacement planning for medical devices. In South Korea, the public procurement service (PPS) has determined basic matters regarding the acquisition, storage, usage, and disposal of national goods. For the efficiency of goods management, the life cycle — an economical usage period — is set, determined by the priority for goods having a large amount of stock and high activity frequency. The life cycle of similar products can be applied to goods without one; in the case of medical devices, the life cycle must be determined for only some of the devices.

### Analysis method

In this study, an empirical life cycle calculation method was utilized to derive the appropriate life cycle for high-risk medical devices. In the case of engineering life cycle calculation methods, data such as accumulated preventive inspection costs and repair costs are required. For high-risk medical devices, the history data of the device were insufficient, so the life cycle of high-risk medical devices was suggested through an empirical life cycle calculation method.

For our analysis, medical device names were matched to compare the mean value of life cycles for the Korean PPS and the AHA with the mean value of the number of used ages for each type of medical device discarded at Samsung Medical Center. In addition, the life cycle data presented by the Korean PPS and the AHA were comparatively analyzed to calculate the mean life cycle. In particular, the life cycle data presented by the AHA were judged to have high reliability because they were obtained by analyzing data accumulated over a long period of time. The life cycle data of the Samsung Medical Center were used to calculate the mean value of the life cycles for each type of medical device discarded at medical institutions. The Samsung Medical Center utilizes approximately 9,600 medical devices of 560 types, replacing old units with new ones through annual deliberation. Among the medical devices in use, the number of high-risk medical devices related to patients' life support was 193 units of seven types, with an average replacement cycle of 14–18 years. The computation of life cycles for high-risk medical devices was made by comparing the mean of life cycles of the Korean PPS and the AHA with the mean of life cycles for each type in Samsung Medical Center; the specific calculation formula is as follows (see Table 1 for further explanation):


(1) $$\begin{array}{l} A=\;\frac{Life\;cycle\;of\;the\;PPS\;+\;Life\;cycle\;of\;the\;AHA\;}{n} \end{array}$$

(2) $$\begin{array}{l} B=\;\frac{Years\;of\;use\;of\;discarded\;devices\;in\;SMC\;}{n} \end{array}$$

(3) $$\begin{array}{l} A\;<B\;=Aug\left\{Aug\left(A,B\right),B\right\},A\;\geq B\;=B \end{array}$$

**Table 1:** Life cycle calculation formula.

**No**	**A**	**Vs**.	**B**	**C**
1	A	<	B	Aug {Aug (A,B),B}
2	A	≥	B	B

A, Average life cycle in Public Procurement Service and the American Hospital Association; B, Average research on usage of medical devices at Samsung Medical Center; C, Recommended life cycle.

## Results

### Medical device regulation in Korea and foreign countries

The US medical device regulatory agency is the Food and Drug Administration (FDA), whose purpose is to protect patients by identifying the safety and effectiveness of medical devices under the United States Department of Health and Human Services (HHS). In particular, the Center for Devices and Radiological Health (CDRH) is the department in charge of most medical devices among the organizations under the FDA, which manages medical devices in general, including registration, reporting, permission, GMP, safety evaluation, follow-up management, etc. (19, 20). The US medical device classification system is classified into Class I, Class II, and Class III according to the risk level, and the higher the grade, the higher the risk level. In addition, according to the criteria for each treatment subject, it is divided into 868 units of anesthesiology department, 872 units of dental department, 882 units of neurology department, and 892 units of radiology, which have a quality assurance system for the design and manufacture of medical devices, similar to ISO 13485.

In the UK, the Medicines and Healthcare Products Regulatory Agency (MHRA) is in charge of all work in terms of certification, licensing, and sales of medical devices (21). In addition, they regulate medical devices through Article 93/42/EEC of the Medical Device Directive (MDD), Article 90/385/EEC of the Active Implantable Medical Device Directive (AIMDD), and Article 98/79/EC of the In vitro-Diagnostic Medical Device Directive (IVDD). Medical device grades in the UK are classified into Class I, Class IIa, Class IIb, and Class III; the higher the grade, the higher the risk level, as in the US.

In Canada, the Medical Devices Bureau of the Therapeutic Products Directorate (TPD) under Health Canada is responsible for regulating the entire life cycle of medical devices by monitoring their safety, effectiveness, and quality before marketing them. In addition, the Marketed Health Products Directorate (MHPD) assesses the safety and riskiness of medical devices after marketing (22). Medical devices are managed in accordance with the Food and Drug Act and Medical Device Regulations. Canada, a member of the International Medical Device Regulators Forum (IMDRF), classifies medical devices into four grades according to the extent of their effects and risks to the human body (23).

In Japan, the Ministry of Health, Labor, and Welfare (MHLW) handles licensing and monitoring work on medical devices as well as regulations on medical devices, *in vitro* diagnostic reagents, and pharmaceuticals. Furthermore, the Pharmaceutical and Medical Device Agency (PMDA), an agency under the MHLW, evaluates the suitability of clinical and non-clinical standards, medical device manufacturing, quality control standards, and systems. Medical devices are classified into four classes based on their importance and risk level according to the Medicine and Medical Appliances Act (24): general medical devices, managed medical devices, designated management medical devices, and advanced-management medical devices. In Japan, according to the characteristics of medical devices, they are managed by dividing them into specific maintenance and installation management medical devices. In the case of specific maintenance medical devices, the license must be renewed every 6 years for quality control.

In Korea, the Ministry of Food and Drug Safety (MFDS) oversees the work related to the safety of medical devices, regulating all matters related to medical devices, including the manufacture, import, and sale of medical devices in accordance with the Medical Appliances Act (25, 26). The Medical Appliances Act provides the basis for the definition, authorization, service, and follow-up management of medical devices. Furthermore, the Medical Appliances Act stipulates and operates matters related to committee operation, follow-up management, etc., whereas the Enforcement Regulations of the Medical Appliances Act stipulate and operate matters related to item approval, quality, clinical tests, etc. The targets regulated by the Medical Appliances Act refer to the medical device handlers and instruments, and the scope of the handlers refers to those who have declared or obtained permission under this Act, including the manufacturer, importer, distributor, or leasing company that handles medical devices for business purposes, the founder of a medical institution under the Medical Law, and the founder of a veterinary clinic under the Veterinarians Act. Medical devices are categorized into four grades according to the usage purpose and the potential hazard degree to the human body when used; the higher the grade, the higher the risk to the medical device. In Korea, it was found that the replacement time is set and managed only for five types of diagnostic radiation generators and 11 types of special medical equipment, according to Articles 37 and 39 of the Medical Law, and that there are no specific regulations on the replacement time and life cycle for other medical devices (see Table 2 for further explanation).

**Table 2:** Risk based classification.

**Nation**	**Class**	**Risk**
US	1	Lowest
	2	Low
	3	Moderate
	4	High
EU	1	Lowest
	2a	Low
	2b	Moderate
	3	High
Canada	1	Lowest
	2	Low
	3	Moderate
	4	High
Japan	1	Lowest
	2	Low
	3	Moderate
	4	High
Korea	1	Lowest
	2	Low
	3	Moderate
	4	High

### Management and replacement of medical devices in Korea and foreign medical institutions

The United States has announced the depreciation lifespan of assets about every 5 years, based on information gathered by the AHA from medical device manufacturers and medical professionals. The replacement plan depending on the lifespan includes the usage frequency, availability of funds, risk factors for safety, etc., and the expected lifespan ranges from at least 5–15 years, most of which were found to be distributed in the 7–10 year range. The lifespan of each medical device was stipulated in the Personal Asset Management Evaluation Guidelines of the Nevada Tax Commission in 2017–2018 as follows: The lifespan of high-tech diagnostic medical devices, such as echocardiography and diagnostic ultrasound scanners, computed tomography devices, magnetic resonance imaging devices, nuclear medicine cameras, positron emission tomography devices, etc., is stipulated as 5 years; that of cardiac laser devices, a high-tech electronic medical device, as 3 years; and that of anesthesia machines, patient-monitoring devices, defibrillators, electrocardiogram (electroencephalogram) devices, cardiac pacemakers, medical laser units, oximeters, spirometers, etc., as 7 years.

In the UK, safety and performance management activities for medical devices are performed by the MHRA, which is in charge of the licensing and regulation management of pharmaceuticals and medical devices. In addition, the MHRA continuously monitors whether devices used in medical institutions have an acceptable level of safety. Furthermore, the imaging service certification system of the UK is responsible for the performance management of imaging diagnosis-based medical devices, while operating a certification program called ISAS, and is running a system through certification of imaging diagnostic medical devices and every-four-year performance testing.

In Canada, the CAMRT announces life cycle guidelines for medical imaging devices and manages them accordingly, for the purpose of helping determine when to upgrade or replace existing devices. According to the guidelines, radiation equipment including roentgenoscopes were stipulated as lasting for 5–10 years, angiography apparatuses as 7 years, computed tomography devices as 7 years, MRI systems and sonography devices as 6 years, mammography equipment as 5–7 years, and single-photon emission computed tomography systems as 10 years.

In Japan, follow-up management, maintenance, and inspection of radiation instruments, such as X-rays, are part of the duties of medical institutions and should be conducted by the institution itself. In addition, according to Articles 9–12 of the Medical Law Matters Regulations, it is possible for a medical institution to entrust the duties to a person (repairer, etc.) recognized as someone who can properly conduct maintenance and inspection. Generally, medical institutions should determine the person in charge of medical devices to make sure that manufacturers and distributors perform designated routine inspections; at the same time, this manager signs repair contracts with repairers and others or commissions regular safety inspections. The quality management system is composed of three categories: quality GMP assurance standards for medical devices, which recommends that they be applied to high-risk medical devices; quality assurance standards at medical device manufacturing facilities, which correspond to the manufacturing and quality-control standards of medical devices; and the manufacturing and quality-control rules of medical devices, which are applied to all medical devices (27).

For evaluation and management of medical device operations, Korean medical institutions comply with matters regarding medical device management stipulated in Chapter 11.5 of the Medical-Institution Evaluation Accreditation Criteria Guidebook of the Korea Institute Healthcare Accreditation, established based on Article 58 of the Medical Law. The key to medical device management is to maintain the performance of medical devices by conducting regular inspections for medical devices and ensuring safety in their use through the prevention of malfunctions. For special medical equipment, it is necessary to conduct regular quality inspections of images. Quality inspection consists of document inspection and on-site inspection; a regular document inspection should be performed every year, and an in-depth document inspection should be conducted every 3 years. The use of devices judged to be inappropriate as a result of inspection is prohibited. Diagnostic radiation generators must undergo electrical and radiation safety inspections every 3 years; however, in the case of relocated equipment, changed power source equipment, and repaired or replaced high-pressure generator, X-ray tube, and control units, irregular inspections should be performed.

### Medical device replacement evaluation criteria

A literature search on domestic and foreign medical-device regulations and medical institutions' medical-device replacement standards revealed no specific life cycle regulations for high-risk medical devices. However, it was found that most medical institutions select evaluation items when replacing and using medical devices, according to the set priorities. Accordingly, in this study, a case analysis and an on-the-spot survey were conducted targeting 10 major domestic and foreign medical institutions to confirm the specific medical device replacing the evaluation criteria. As a result of the analysis, there were fewer than 10 evaluation items, and the evaluation items for medical device replacement and the mark-distribution table for each item are shown in Table 3.

**Table 3:** Evaluation items and scoring table.

**No**	**Evaluation item**		**SMC** ** (Korea)**	**AUMC** ** (Korea)**	**KUMC** ** (Korea)**	**Winnipeg** ** (Canada)**	**Hamilton** ** (Canada)**	**Umass** ** (US)**	**U.S.Military** ** (US)**	**Stanton** ** (Canada)**	**VA** ** (USA)**	**Giza** ** (Egypt)**
1	Failure rate		25	10	10	10		O	70.35			
2	Maintenance cost rate		10	10		20	5.4	O	8.04			O
3	Model discontinuation		10	30	30							
4	Part discontinuation		15				16.1	O		x2		O
5	Age		20	25	20	10	13.6	O	4.02	5	O	O
6	Daily inspection		4									
7	BMET evaluation		16	25	20							
9	Reliability					50						
10	Device obsolescence						18.9			5		
11	Frequency of use				10		15.4					
12	Repair time						4.6					O
13	Equipment risk				10	10	20.7					
14	Purchase amount						5.4					
15	Model unity											
16	Accident history							O		x4		O
17	Technological progress							O	7.04		O	
18	5-year plan								10.55			
19	Physical high risk			10						5	O	
20	Service response time											O
21	Use of backup devices			10						x2		
22	Main device (expensive)									5		
23	Adjustment	Obsolescence			20							
24	variable	Depreciation			x0.5, x1					x0.5		
25		Accident history			x3							
Highest point			100	120	320	100	100	Item disclosure	100	50	Some disclosure	Item disclosure

SMC, Samsung Medical Center; AUMC, Ajou University Medical Center; KUMC, Konkuk University Medical Center; BMET, biomedical equipment technician.

In addition, the results of deriving the top 10 factors by assigning weights according to the frequency of evaluation items are shown (Table 4). The most important items were age (15.87%), followed by maintenance cost (11.11%), partial discontinuation (11.11%), failure rate (9.52%), high physical risk (6.35%), model discontinuation (4.76%), BMET evaluation (4.76%), equipment risk (4.76%), accident history (3.17%), and device obsolescence (3.17%).

**Table 4:** Weight ranking by item.

**No**	**Evaluation item**	**Frequency**	**Weight (%)**	**Ranking**
1	Age	10	15.87%	1
2	Maintenance cost rate	7	11.11%	2
3	Part discontinuation	7	11.11%	2
4	Failure rate	6	9.52%	4
5	Physical high risk	4	6.35%	5
6	Model discontinuation	3	4.76%	6
7	BMET evaluation	3	4.76%	6
8	Equipment risk	3	4.76%	6
9	Accident history	2	3.17%	9
10	Device obsolescence	2	3.17%	9

### Life cycle calculation of high-risk medical devices

To calculate the life cycle of high-risk medical devices, we intended to identify the life cycle of the Korean PPS and the AHA and then derive the appropriate life cycle by using the empirical life cycle calculation method. First, by comparing the life cycle standards of 28 medical-device items according to the Commodity Management Act of the Korean PPS with those of the AHA, it was found that the life cycle in Korea was longer in the case of imaging equipment, such as CT and MRI, than in the US (Table 5).

**Table 5:** Comparison of the life cycle in medical devices in Korea and US.

**No**	**Name**	**PPS (Korea)**	**AHA (US)**
1	CT	10	7
2	DNA sequencer	10	7
3	ECG, portable	10	7
4	MRI	10	7
5	Pure water system	10	7
6	Spectrophotometer	11	8
7	Ultrasound image system	10	7
8	Ventilator	10	7
9	Spectrometer, LC/MS	10	8
10	Analyzer, chemistry	9	8
11	X-ray system	11	10
12	Centrifuge, refrigerated	8	8
13	Defibrillator, W/MONITOR	7	7
14	Hemodialysis apparatus	7	7
15	HPLC	7	7
16	Water bath	10	10
17	Anesthesia machine	9	10
18	Audiometer	9	10
19	Freezer, deep	9	10
20	Infusion pump	9	10
21	Stainer, auto	9	10
22	Electrosurgical unit	8	10
23	Sterilizer, steam	10	12
24	Microscope, binocular	12	15
25	Slit lamp	8	12
26	C.P.M KNEE	9	15
27	Surgical light	9	15
28	Heart-lung machine	-	8

AHA, The American Hospital Association; PPS, Public Procurement Service.

Next, a comparative analysis was conducted on the life cycle data of the Korean PPS and the AHA; as a result, in the case of the same device, a total of 878 devices with life cycle data were found on either side. Specifically investigating, cases in which the standards of the AHA standard had a shorter life cycle than that of the Korean PPS appeared to include 663 units (76%). In addition, the standard of the Korean PPS had a shorter life cycle than that of the AHA at 31 units (4%), while the case was 13 units (1%), showing that the life cycle standards of both the Korea PPS and the AHA were the same. The case with only the life cycle standards of the AHA appeared to be 109 units (12%), whereas the case with only those of the Korean PPS was found to be 49 units (6%), and the case without any life cycle standards for both the Korea Procurement Service and the AHA was 13 units (1%) (Table 6).

**Table 6:** Life cycle standard in Korea and US.

**Period of durability**	**PPS (Korea)**	**Vs**.	**AHA (US)**	**Ratio (%)**	**Count**
1	A	>	B	76%	663
2	A	<	B	4%	31
3	A	=	B	1%	13
4	X		B	12%	109
5	A		X	6%	49
6	X		X	1%	13
Total				100%	878

Based on this, the life cycle means for the Korea PPS and the AHA was derived, and the proper life cycle for high-risk medical devices was calculated by comparing this to the usage research mean for each type in Samsung Medical Center. Based on such a standard, the proper life cycle of four high-risk medical devices calculated by empirical methods appeared to be 13 years for anesthesia machines, 14 years for defibrillators, 16 years for heart-lung machines, and 13 years for ventilators (Table 7).

**Table 7:** An appropriate life cycle for high-risk medical devices.

**Medical devices**		**PPS, AHA Average**	**SMC average used period**	**An appropriate life cycle**
1	Anesthesia machine	8	14	13
2	Defibrillator	6	17	14
3	Heart-lung machine	8	18	16
4	Ventilator	10	14	13

## Discussion

In this study, we intended to derive the life cycle of high-risk medical devices through empirical methods, based on the life cycles of the Korea PPS and the AHA, and on the mean period used for the replaced devices of the Samsung Medical Center. Thus, the proper life cycle of high-risk medical devices related to life support was calculated as 13 years for anesthesia machines, 14 years for defibrillators, 16 years for heart-lung machines, and 13 years for ventilators. Medical devices are devices that perform invasive functions in the patient. Therefore, management must be performed on the devices, as risk occurs in the medical device itself, as well as through the carelessness of users. As performance degradations or quality abnormalities, caused by insufficient management of medical devices, may induce direct harm to patients, it seems that the replacement at the right time through life cycle management will be able to prevent harm. In particular, because medical devices, such as life-support machines, belong to the high-risk medical devices and thus are included in the priority management target, the life cycle management of these devices must be performed first.

In general, for the replacement of medical devices, various factors must be considered, such as life expectancy, priorities for each set item, safety and efficiency of devices, risk evaluation, government policies (radiation safety, etc.), expectations for potential profits, management policy judgments, etc., including the performance and operating costs of the device. In particular, in an environment where life cycle management is not conducted to replace medical devices, there is a limit to replacing medical devices using objective evaluation methods (28). In this study, as a result of analyzing the evaluation items on the life cycle of medical devices, the importance of age, maintenance cost, part discontinuation, failure rate, and physical high risk appeared to be the highest, and it seems that the selection of the managing target device should be performed based on this.

Currently, the life cycle evaluation of medical devices is designed to allow the user to generate the replacement priority result of the device in real time, where the criteria, standards, and guides are utilized in the clinical, technological, manpower, patient, economic, and institutional aspects for medical device evaluation. There are two evaluation methods for preparing and utilizing a score table for the degree of deterioration and making decisions through a committee. As a result of conducting a literature survey on the replacement regulations of medical devices, we found that there is no standard for the replacement of medical devices prescribed by the country or regulatory agency, and it was found that the items on the replacement standards were different for each medical institution. In order to overcome these limitations, it will be necessary to reorganize the management system through the role allocation of the government and medical institutions. At the government level, it is necessary to exercise authority over regulations by establishing management standards through the reorganization of legislation and systems related to medical device replacement. However, the application of the same policy level to all medical devices is realistically limited; therefore, it is necessary to take a step-by-step approach through the appropriate classification system. Furthermore, medical institutions must establish and operate standards for the life cycle considering the characteristics of medical devices through expert organizations. Thus, if the medical device is operated safely in the optimal state, the medical institution could provide high-quality medical services and contribute to patient safety.

## Conclusion

In the case of the life cycle calculation by the empirical methods presented in this study, there is a limit within which deviations may occur, according to the characteristics of each medical institution, on-site conditions, etc. Therefore, it is necessary to simultaneously utilize both engineering and empirical methods to calculate life cycles. To achieve this, it is necessary to accumulate a database with careful history management so that the history of medical devices may not be missing. In future research, it will be possible to derive replacement decision factors for all medical devices and calculate the appropriate life cycle of medical devices by applying engineering and empirical methods together. Nevertheless, in this study, we calculated the proper life cycle of high-risk medical devices as the priority management target; based on this, the annual plan for medical devices seems to be enabled. Moreover, performing the planned replacement of medical devices will reduce the risk to patients due to the deterioration of devices and have a positive impact on the management performance of medical institutions by estimating the annual budget for purchasing.

## Data availability statement

The data analyzed in this study is subject to the following licenses/restrictions: The datasets presented in this article are confidential and not readily available for use by Samsung Medical Center. Requests to access these datasets should be directed to Gihong Seo, lionsgh@skku.edu.

## Author contributions

GS contributed to the conception, data collection, data analysis, and writing—original draft. SP contributed to the design of the study and editing of the original and revised manuscripts. ML supervised the entire process of the development and revision of the manuscript. All authors contributed to manuscript revision, read, and approved the submitted version.

## Funding

This work was supported by the Ministry of Education of the Republic of Korea and the National Research Foundation of Korea (NRF-2021R1I1A4A01057428) and Bio-convergence Technology Education Program through the Korea Institute for Advancement Technology (KIAT) funded by the Ministry of Trade, Industry and Energy (No. P0017805).

## Conflict of interest

The authors declare that the research was conducted in the absence of any commercial or financial relationships that could be construed as a potential conflict of interest.

## Publisher's note

All claims expressed in this article are solely those of the authors and do not necessarily represent those of their affiliated organizations, or those of the publisher, the editors and the reviewers. Any product that may be evaluated in this article, or claim that may be made by its manufacturer, is not guaranteed or endorsed by the publisher.
